# Expression of the neuroprotective factors BDNF, CNTF, and FGF-2 in normal and oxygen induced retinopathy

**DOI:** 10.3389/fnins.2022.971952

**Published:** 2022-12-02

**Authors:** Jifu Xin, Yuhong He, Kai Guo, Dayong Yang

**Affiliations:** Department of Ophthalmology, The Affiliated Hospital of Inner Mongolia Medical University, Hohhot, China

**Keywords:** brain-derived neurotrophic factor, ciliary neurotrophic factor, fibroblast growth factor 2, ischemic-induced retinopathy, OIR, retina, neuroprotection

## Abstract

**Introduction:**

Oxygen-induced retinopathy is a type of retinal pathological neovascularization (NV) disease that leads to vision loss and translates to a significant societal cost. Anti-vascular endothelial growth factor (VEGF) and anti-inflammatory treatments have been widely used in the clinic, but the results have not been entirely satisfactory. It is necessary to explore other treatments for Ischemic retinal diseases.

**Methods:**

The oxygen-induced retinopathy (OIR) model was induced from P7 to P12 as described. Histology evaluation (HE) and retina flat mounts were checked at P17 to confirm the establishment of the OIR model. Retinal ganglion cell (RGC) degeneration was checked by transmission electron microscopy at P17 to confirm the neurological damage caused by OIR. Western blot analysis was performed at P12, P15, and P17 to study the expression of brain-derived neurotrophic factor (BDNF), ciliary neurotrophic factor (CNTF), and fibroblast growth factor 2 (FGF-2) in normal and OIR mice. Comparative analysis of the expressions of BDNF, CNTF, and FGF-2 in normal and OIR mice was performed.

**Results:**

There were many retinal NV and non-perfusion areas in OIR P17. RGCs were degenerated at OIR P17. The expressions of BDNF, CNTF, and FGF-2 gradually increased from P12 to P17 in normal mice and were much higher in OIR mice. The expression curves of BDNF, CNTF, and FGF-2 in the OIR model were inconsistent and did not correlate with each other.

**Discussion:**

This study provides evidence for changes in BDNF, CNTF, and FGF-2 in Oxygen-induced retinopathy.

## Introduction

Ischemic-induced retinopathy is a type of retinal pathological neovascularization (NV) disease induced by retinal ischemia and hypoxia, and its progressive and irreversible nature leads to vision loss and translates to a significant societal cost (Brown et al., 2003; Hellström et al., 2013). Antioxidative drugs, gene therapy, and cell-based therapies are being investigated to reduce the rate of disease progression, but the clinical treatment results are still not entirely satisfactory. Exploring better treatment strategies not limited to anti-vascular endothelial growth factor (VEGF) or anti-inflammatory agents is essential.

The primary pathological damage mechanism of ischemic-induced retinopathy is an imbalance in the production of reactive oxygen species (ROS) and the activity of biological detoxifying systems, which eventually leads to a change in cell structure and functionality through the alteration and degradation of molecules (Calderon et al., 2017). Studies have shown that ischemic retinopathy affects astrocytes, retinal neurons, glial cells, and Muller cells, in addition to vascular endothelial cells (Akula et al., 2007; Lofqvist et al., 2007; Ng et al., 2016; Coughlin et al., 2017; Filippi and Dal Monte, 2020). Protecting retinal neurotrophic functions has become a treatment strategy; however, more experimental data is required to support its use. Neuroprotective functions are mediated by neurological factors, including brain-derived neurotrophic factor (BDNF), ciliary neurotrophic factor (CNTF), and fibroblast growth factor 2 (FGF-2) (LaVail et al., 1992). Several reports confirmed that BDNF, CNTF, and FGF-2 could inhibit the degeneration of retinal function (Qin et al., 2011; Laughter et al., 2018; Valiente-Soriano et al., 2019). This study used the oxygen-induced retinopathy (OIR) model, a classical animal model of ischemic-induced retinopathy, to investigate and analyze the expression change and correlation of BDNF, CNTF, and FGF-2 to provide theoretical data for further research on neuroprotective therapy.

## Materials and methods

### Oxygen-induced retinopathy model and animals

Pregnant C57BL/6J mice were provided by the Laboratory Animal Center of Southern Medical University, China. All experiments adhered to the Association for Research in Vision and Ophthalmology Statement for the Use of Animals in Ophthalmic and Vision Research and were approved by the Institutional Animal Care and Use Committee of Inner Mongolia Medical University, China. The OIR model was performed as described by Smith et al. (1994). Seven-day-old (P7) mice pups with their mother were exposed to 75 ± 2% oxygen for 5 days (P7–P12). Then, P12 mice were transferred to room air until P17. Mouse pups were maintained in a normal environment as the wild-type (WT) control group. The nursing mothers were rotated from high oxygen to room air every 24 h to prevent oxygen toxicity.

### Histology evaluation

Enucleated eyes obtained after euthanasia-induced sacrifice were fixed with 4% neutral paraformaldehyde for 24 h, then dehydrated by gradient ethanol, transparent xylene, and embedded in paraffin wax. The retina was continuously sliced at a thickness of 4 μm parallel to the sagittal axis of the optic nerve. The slices were baked overnight, deparaffinized with xylene, hydrated with gradient ethanol, stained with hematoxylin and eosin, and then observed under a microscope (Zeiss Axioplan 2 imaging, Gottingen, Germany). One slice was randomly selected from every five slices, and a total of five slices were observed from each eye. The vascular endothelial cell (VEC) nucleus that broke through the retina inner limiting membrane (ILM) was counted under 10X magnification in one slice. The HE of retinas from the WT control and OIR groups were examined at P17.

### Retinal flat mount

Mouse pups were anesthetized and retro-orbitally injected with fluorescein isothiocyanate dextran as described by Li et al. (2011). Then, after 10 s, mice were euthanized using pentobarbital. Enucleated eyes were fixed in 4% paraformaldehyde for 30 min at room temperature and then washed three times in phosphate-buffered saline (PBS). The retinas were separated from the sclera, retinal pigmented epithelium, lens, cornea and cut into four parts. Water-soluble mounting tablets were used for mounting, and coverslips were added. The retinal flat mounts were photographed at 5 × original magnification with fluorescence microscopy (Zeiss Axioplan 2 imaging). The combined exposure time of the retinal flat mounts was 1.5 s. The retinal segments were merged to generate an image of the total retina (Photoshop 2020; Adobe Systems Inc., San Jose, CA, United States) and the non-perfusion area was analyzed (Connor et al., 2009). Flat-mounted retinas from the WT control and OIR groups were examined at P17.

### Transmission electron microscopy

Enucleated eyes were fixed in 2.5% glutaraldehyde at 4°C overnight, then in 1% osmium tetroxide at room temperature, and dehydrated in ethanol. After incubating in acetone for 20 min, the eyes were treated with 50% (1 h), 75% (3 h), and 100% (overnight) epoxy resin and heated at 70°C overnight. The eyes were sliced to sections of about 70 nm. One slice was randomly selected from every five slices, and a total of three slices were observed from each eye. Sections were stained with 3% uranyl acetate and 3% lead citrate for 15 min at room temperature and then observed by electron microscopy. Retinal ganglion cells (RGC) from the WT control and OIR groups were examined by electron microscopy at P17.

### Western blotting analysis

According to the manufacturer’s instructions, the total protein concentration of retinal extracts (four retinas of four mice per condition) was measured using a Protein Quantification Kit. The proteins were eluted with Laemmli buffer, subjected to 12% sodium dodecyl-sulfate polyacrylamide gel electrophoresis (SDS-PAGE), then transferred to polyvinylidene fluoride membranes. The membranes were blocked with Tris-buffered saline containing 0.1% Tween-20 (TBST) and 5% dried non-fat milk at room temperature for 2 h. After washing with TBST three times (10 min), the membrane was incubated with a primary antibody diluted in blocking solution overnight at 4°C. The primary antibodies used in our study were anti-BDNF antibody (1:500, Santa Cruz Biotechnology, TX, USA, sc-546), anti-CNTF antibody (1:200, Santa Cruz Biotechnology, TX, USA, sc-365210), anti-FGF-2 antibody (1:200, Santa Cruz Biotechnology, TX, USA, sc-74412), and anti-beta Actin antibody (1:5000, Abcam, MA, USA, ab6276). After incubation, the membrane was washed three times with TBST for 10 min and incubated with anti-rabbit IgG F(ab′)2 secondary antibody (1:1000, Invitrogen, CA, USA, 31461) for 2 h at room temperature. Finally, the immunoreactive bands were developed by enhanced chemiluminescence and detected using photographic film. The protein expressions of BDNF, CNTF, and FGF-2 from the WT control and OIR groups, were examined at P12, P15, and P17.

### Statistical analyses

Data were reported as the mean ± standard deviation (SD). Statistical analysis was performed using statistical software (SAS Institute Inc., Cary, NC, United States). Data were analyzed using a *t*-test to compare the differences between two groups at different time points. Pearson’s correlations were used to compare the correlation of various factors in the same group. *P* < 0.05 was accepted as statistically significant.

## Results

### Retina neovascularization and non-perfusion areas were present in oxygen-induced retinopathy mice

**FIGURE 1:**
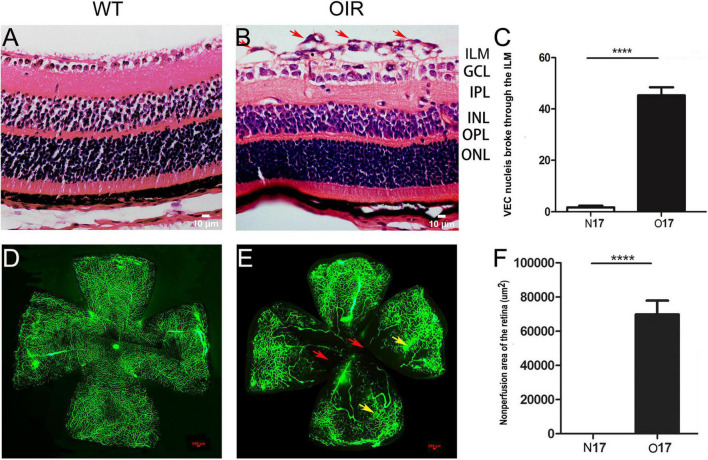
P17 oxygen-induced retinopathy (OIR) mice developed retina neovascularization (NV) and non-perfusion areas. **(A)** Representative histology evaluation (HE) of retinal sections from the wild-type (WT) control group. A few vascular endothelial cell (VEC) nuclei and new blood vessels broke through the retina ILM. **(B)** Representative HE of retinal sections from the OIR group. There were many VEC nuclei and new blood vessels that broke through the retina ILM (red arrows). **(C)** The number of VEC nuclei in the OIR group that broke through the retina ILM was significantly higher than in the WT control group. **(D)** Representative retinal flat mount image from the WT control group. There were no NV and non-perfusion areas. **(E)** Representative retinal flat mount images from the OIR group. There were many NV (yellow arrows) and non-perfusion areas (red arrows). **(F)** The area of non-perfusion in the OIR group was significantly higher than in the WT control group. Nuclei numbers and non-perfusion areas were calculated using Image-Pro Plus software. ****P < 0.0001 vs. WT control group. ILM, inner limiting membrane; GCL, Ganglion cell layer; IPL, inner plexiform layer; INL, inner nuclear layer; OPL, outer plexiform layer; ONL, outer nuclear layer.

Retina NV and non-perfusion areas were used to confirm the successful establishment of the OIR model. VEC nuclei and new blood vessels that broke through the retina ILM were rarely seen in the P17 WT control group (Figure 1A) (1.70 ± 0.68; Figure 1C). However, significantly higher numbers of VEC nuclei and new blood vessels broke through the retina ILM in the P17 OIR group (Figure 1B) (45.30 ± 3.13; Figure 1C), compared with the control group (*t* = 86.5, *P* = 0.00). The retinal flat mount of the P17 WT control group showed that large blood vessels were smooth with distributed capillaries, and no avascular and non-perfusion areas (Figure 1D). In the OIR group, the large blood vessels had a tortuous expansion, massive neovascular fluorescence appeared in the periphery, the capillary network structure disappeared, and there were massive NV and non-perfusion areas (Figure 1E). The area of non-perfusion in the OIR group was significantly higher than that in the WT control group (*P* < 0.0001) (Figure 1F).

### Degeneration of retinal ganglion cell in the oxygen-induced retinopathy group confirmed by electron microscopy

**FIGURE 2:**
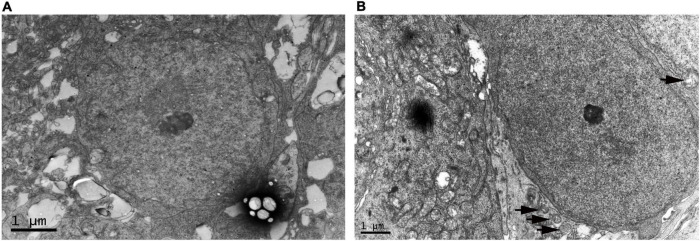
Electron microscopy of retinal ganglion cell (RGC) in the P17 wild type (WT) control and oxygen-induced retinopathy (OIR) groups. **(A)** Representative electron microscopy images of the WT control group. RGC had defined plasma membranes and uniformly distributed chromatin. **(B)** Representative electron microscopy in the OIR group. The cytoplasm of RGC was swollen, the nuclei of RGC were darkened, and autophagosomes had appeared to increase (black arrow).

We compared RGC in the WT control group and OIR group at P17 by electron microscopy to confirm the neurological damage caused by ischemic-induced retinopathy. We found that RGC had defined plasma membranes and uniformly distributed chromatin in the WT control group (Figure 2A). However, in the OIR group, mitochondria and cytoplasm were swollen, the nuclei of RGC were darkened, and autophagosomes had appeared to increase, suggesting that RGC degeneration had occurred in OIR mice at P17 (Figure 2B).

### Expression of brain-derived neurotrophic factor, ciliary neurotrophic factor, and fibroblast growth factor 2 in the wild-type control and oxygen-induced retinopathy groups

**FIGURE 3:**
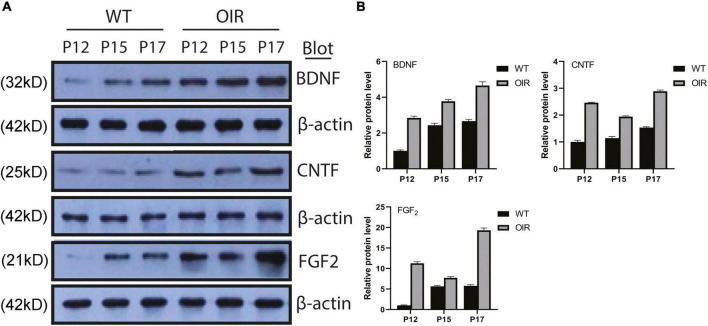
Expressions of BDNF, CNTF, and FGF-2 in the WT control and OIR groups. **(A)** Western blot analysis of the protein expressions of BDNF, CNTF, and FGF-2 in the WT control and OIR groups at P12, P15, and P17. The relative protein expression levels were normalized to β-Actin. **(B)** Quantification revealed trends in the expressions of BDNF, CNTF, and FGF-2 in the WT control and OIR groups at P12, P15, and P17. BDNF, brain-derived neurotrophic factor; CNTF, ciliary neurotrophic factor; FGF-2, fibroblast growth factor 2; WT, wild type; OIR, oxygen-induced retinopathy.

The expression of BDNF, CNTF, and FGF-2 protein increased gradually from P12 to P17 in the OIR group, similar to the WT control group (Figure 3A). The expression of BDNF, CNTF, and FGF-2 in the OIR group was statistically higher than in the WT control group at P12, P15, and P17, respectively. (BDNF: *P*-values: < 0.0001, < 0.0001, < 0.0003; CNTF: *P*-values: < 0.0001, < 0.0001, < 0.0001; and FGF-2: *P*-values: < 0.0001, < 0.0001, < 0.0001, respectively) (Figure 3B). Furthermore, the expression of CNTF and FGF-2 in the OIR group was decreased at P15 (Figure 3B).

### Correlation of different factors during wild-type control and oxygen-induced retinopathy group growth

**FIGURE 4:**
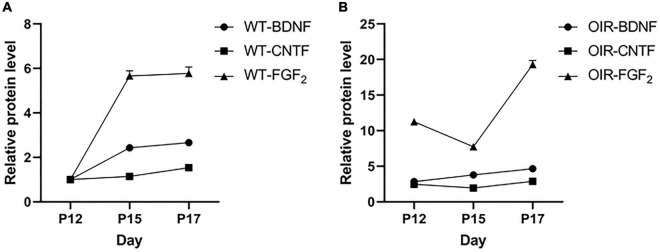
Variation between brain-derived neurotrophic factor (BDNF), ciliary neurotrophic factor (CNTF), and fibroblast growth factor 2 (FGF-2) in the wild type (WT) control and oxygen-induced retinopathy (OIR) groups. **(A,B)** Data from the western blot assay showed the time course of BDNF, CNTF, and FGF-2 expressions at P12–P17 in the **(A)** WT control group and **(B)** OIR group.

The results showed BDNF, CNTF, and FGF-2 in the WT control group (Figure 4A) and OIR group (Figure 4B). There were no strong positive correlations between BDNF, CNTF, and FGF-2 during the growth of normal and OIR mice (Table 1).

**TABLE 1:** Correlation between brain-derived neurotrophic factor (BDNF), ciliary neurotrophic factor (CNTF), and fibroblast growth factor 2 (FGF-2) in the wild type (WT) control and oxygen-induced retinopathy (OIR) groups.

	CNTF		FGF-2	
		
Control group	Pearson’s *r*	*P*-value	Pearson’s *r*	*P*-value
BDNF	0.789	0.421	0.994	0.070
CNTF			0.717	0.491
**OIR group**				
BDNF	0.431	0.717	0.664	0.538
CNTF			0.961	0.179

There were no strong positive correlations between BDNF, CNTF, and FGF-2 during the growth of normal and OIR mice.

## Discussion

The mechanism of ischemic-induced retinopathy disease is complicated and not only related to VEGF. Some clinical cases reflect the limitation of the treatment of anti-VEGF and anti-inflammation (Massin et al., 2010; Wallsh and Gallemore, 2021). This study was designed to provide theoretical data on neuroprotective therapy for ischemic-induced retinopathy. The OIR model is a classic animal model for the study of ischemic-induced retinopathy diseases. It develops pathological retina blood vessels and no perfusion areas that can simulate neurological damage caused by ischemic-induced retinopathy diseases. We checked the HE and flat mounts of the retina to confirm that the OIR model was established successfully (Liegl et al., 2019) and electron microscopy to confirm the neurological damage caused by ischemic-induced retinopathy. Finally, we checked the expression of neuroprotective factors, BDNF, CNTF, and FGF-2 in the retina during the growth of normal and OIR mice to analyze their effects. The results from this study provide evidence for changes in the expressions of BDNF, CNTF, and FGF-2 in Oxygen-induced retinopathy.

Brain-derived neurotrophic factor is a very important factor in ocular development (Zafra et al., 1990). Some studies also showed that BDNF had a neuroprotective role in retina diseases (Behl and Kotwani, 2017; Feng et al., 2017). Seki et al. (2003) confirmed that BDNF protein levels were increased between P14 and 1 M in normal mice retina and remained relatively stable after adulthood. We found that the expression of BDNF gradually increased from P12 to P17 in normal mice, which is similar to the report by Seki et al. (2003). Cheng et al. (2016) reported that the expression of BDNF in OIR C57BL/6J mice on P17 was significantly higher than in normal mice. Our study not only confirmed the expression of BDNF in P17 OIR mice was higher than in P17 normal mice but also showed the BDNF expression in OIR mice between P12 to P17 ware increased gradually and linearly and was statistically higher than in normal mice at each time point.

Ciliary neurotrophic factor, a neuropoietic cytokine that belongs to the IL-6 family (Bauer et al., 2007), exerts a neuroprotective effect on photoreceptors and RGCs by stimulating regeneration (Wen et al., 2012; Wang et al., 2018). A previous study showed the levels of CNTF RNA in normal mice at P12, P14, and P17 were similar (Bucher et al., 2016). In contrast, another study of Cao et al. (2001) showed that CNTF RNA in normal mice increased after birth, reached a maximum around P120, and tended to balance afterward. Our results showed that the expression of CNTF was increased in normal mice from P12 to P17, similar to that in Cao et al. (2001). Dorfman et al. (2009) reported that CNTF levels in Sprague-Dawley (SD) and Long Evans (LE) rats appeared higher after exposure to hyperoxic from P6 to P14 compared with their corresponding controls (Dorfman et al., 2009). They also confirmed that the outer nuclear layer (ONL), outer plexiform layer (OPL), inner nuclear layer (INL), and inner plexiform layer (IPL) of the OIR rat were statistically thinner than the normal rat. We showed that the expression of CNTF in OIR mice was higher than in normal mice at P12, P15, and P17, similar to results from several studies showing that retinal CNTF levels were upregulated in response to injury (Cao et al., 1997; Lipinski et al., 2011). We analyze that the high expression of CNTF may be self-protection under pathological conditions. In addition, our data show that the CNTF expression curve did not increase linearly and that the lowest expression was at P15 in the OIR group, suggesting an increased consumption of CNTF in P15.

Fibroblast growth factor 2, expressed by photoreceptors, other retinal cells, and the central nervous system (Raballo et al., 2000; Marticorena et al., 2012), enhances endothelial cell proliferation, migration, and survival (Gao and Hollyfield, 1995). Studies have confirmed that FGF-2 could improve visual function in ischemia (Yamada et al., 2001; Lange et al., 2007). Our data showed that the expression of FGF-2 increased from P12 to P17 in the normal and OIR mice. The expression curve in the OIR group decreased in the early stage and increased significantly in the later stage and was much higher than that control group at each time point. Our results are similar to a report by Fang et al. (2015) that found that FGF-2 mRNA was increased, revealing time-dependent effects in control and OIR animals from P8 to P17. However, Dorfman et al. (2009) reported that FGF-2 levels in SD and LE rats tended to increase after exposure to hyperoxic from P6 to P14 but did not reach statistical significance compared with controls. Combined with other studies which confirmed that FGF-2 expression is increased in pathological conditions (Riva et al., 1992; Dong et al., 2019), we thought the different results from Dorfman with us might be related to the tolerance and response to hypoxia differences between rats and mice.

We also investigated a potential correlation between the expressions of BDNF, CNTF, and FGF-2 by Pearson’s correlations analysis in normal and OIR mice, to determine whether there were any direct interactions. Our results indicated no statistical correlations between BDNF, CNTF, and FGF-2 in normal and OIR mice. However, an *in vitro* study showed after being treated with BDNF to mouse Müller glia, the BDNF, CNTF, and FGF-2 have simultaneous upregulation (Harada et al., 2002). We speculate that the different results might be caused by complex mechanisms in the *in vivo* environment.

To our knowledge, this is the first study to simultaneously assess the spatiotemporal changes of the three neural factors in the OIR mice and reveal no correlation in the expression of the three neural factors. It provides theoretical data for neuroprotective treatment for OIR. It suggests that intervening in neurotrophic factor expression in OIR at different time points might be useful. The limitation of this study is that it did not use neural factors to confirm the protective function of the retina. There are still many challenges. One is that neurotrophic factors are hydrophilic and cannot easily pass through the blood-brain or blood-retinal barriers. Second, neurotrophic factors have short half-lives and poor pharmacokinetics *in vivo* (Thorne and Frey, 2001). Furthermore, how to determine the patient’s condition for neuroprotective treatment at the earliest stage of disease, the difference between clinical individual assessment and treatment, combination therapy, and related issues are also significant. More research is needed to answer these questions.

## Data availability statement

The raw data supporting the conclusions of this article will be made available by the authors, without undue reservation.

## Ethics statement

This animal study was reviewed and approved by the Institutional Animal Care and Use Committee of Inner Mongolia Medical University, China. All experiments adhered to the Association for Research in Vision and Ophthalmology Statement for the Use of Animals in Ophthalmic and Vision Research. Written informed consent was obtained from the owners for the participation of their animals in this study.

## Author contributions

JX, KG, and DY performed experiments and data analysis. JX, YH, KG, and DY planned the research, discussed the data analysis, and wrote the manuscript. All authors approved the final version of the manuscript to be published.
